# Progranulin Gene Variability and Plasma Levels in Bipolar Disorder and Schizophrenia

**DOI:** 10.1371/journal.pone.0032164

**Published:** 2012-04-10

**Authors:** Daniela Galimberti, Bernardo Dell'Osso, Chiara Fenoglio, Chiara Villa, Francesca Cortini, Maria Serpente, Sarah Kittel-Schneider, Johannes Weigl, Maria Neuner, Juliane Volkert, C. Leonhard, David G. Olmes, Juliane Kopf, Claudia Cantoni, Elisa Ridolfi, Carlotta Palazzo, Laura Ghezzi, Nereo Bresolin, A. C. Altamura, Elio Scarpini, Andreas Reif

**Affiliations:** 1 Department of Neurological Sciences, University of Milan, IRCCS Fondazione Cà Granda, Ospedale Maggiore Policlinico, Milan, Italy; 2 Bipolar Disorder Program, Department of Psychiatry, Psychosomatics and Psychotherapy, University of Würzburg, Würzburg, Germany; University of Illinois-Chicago, United States of America

## Abstract

Basing on the assumption that frontotemporal lobar degeneration (FTLD), schizophrenia and bipolar disorder (BPD) might share common aetiological mechanisms, we analyzed genetic variation in the FTLD risk gene progranulin (*GRN*) in a German population of patients with schizophrenia (n = 271) or BPD (n = 237) as compared with 574 age-, gender- and ethnicity-matched controls. Furthermore, we measured plasma progranulin levels in 26 German BPD patients as well as in 61 Italian BPD patients and 29 matched controls.

A significantly decreased allelic frequency of the minor versus the wild-type allele was observed for rs2879096 (23.2 versus 34.2%, *P*<0.001, OR:0.63, 95%CI:0.49–0.80), rs4792938 (30.7 versus 39.7%, *P* = 0.005, OR: 0.70, 95%CI: 0.55–0.89) and rs5848 (30.3 versus 36.8, *P* = 0.007, OR: 0.71, 95%CI: 0.56–0.91). Mean±SEM progranulin plasma levels were significantly decreased in BPD patients, either Germans or Italians, as compared with controls (89.69±3.97 and 116.14±5.80 ng/ml, respectively, versus 180.81±18.39 ng/ml *P*<0.001) and were not correlated with age.

In conclusion, *GRN* variability decreases the risk to develop BPD and schizophrenia, and progranulin plasma levels are significantly lower in BPD patients than in controls. Nevertheless, a larger replication analysis would be needed to confirm these preliminary results.

## Introduction

Mutations in the progranulin gene (*GRN*) are the most frequent cause of autosomic dominant frontotemporal lobar degeneration (FTLD). Age at disease onset, as well as clinical phenotypes associated with such mutations are extremely wide, even in the same family [1] and include, besides the classical FTLD syndromes behavioural variant of frontotemporal dementia (bvFTD), progressive non fluent aphasia and semantic dementia [2]–[4], additional presentations such as corticobasal syndrome and progressive supranuclear palsy (PSP). In 2009, Velakoulis et al. [5] presented a post mortem study of young patients, diagnosed ante mortem with psychiatric illnesses including bipolar disorder (BPD) and schizophrenia, and demonstrated the presence of protein deposits [tau or TAR DNA Binding Protein (TDP)43] typical of FTLD brains. Moreover, genetic analysis in one case revealed a *GRN* mutation. Additional evidence of a clinical overlap between psychiatric disorders and genetically determined FTLD comes from the recent description of a patient with heterosexual pedophilia [6] who was a carrier of a *GRN* mutation and developed bvFTD over time, and from a second article reporting two clinically different, apparently sporadic FTLD cases sharing the previously described Thr272fs *GRN* mutation, who had had a premorbid BPD history [7]. Basing on the assumption that FTD and schizophrenia might have a common aetiology in some families in which both syndromes coexist, Schoder et al. [8] analyzed the morbid risk for schizophrenia in first-degree relatives of 100 FTD probands and compared it with first-degree relatives of 100 Alzheimer's disease (AD) relatives, showing that the morbid risk for schizophrenia was significantly higher in relatives of FTD probands than in relatives of AD probands. Notably, in one family, a mutation in *GRN* was found [8]. A major contribution to achieve a correct diagnosis independent of the phenotypic presentation is the demonstration that progranulin plasma levels are extremely low in *GRN* mutation carriers [1], [9]–[11].

Besides autosomic dominantly inherited *GRN* mutations, a contribution of *GRN* genetic variability has been previously shown in sporadic FTLD as well [12], even though another study did not confirm these data [13]. A further association analysis demonstrated that a single nucleotide polymorphism (SNP) in the *GRN* promoter influences the risk for FTLD [14]. Besides the susceptibility effect, *GRN* polymorphisms likely influence gene expression. In this regard, Fenoglio et al. [15] demonstrated that rs5848 *TT* genotype is associated with decreased *GRN* expression levels in brains and peripheral blood mononuclear cells (PBMC) from patients with AD. *GRN* is localized in a region of chromosome 17q21 previously shown to be associated with BPD [16], [17] and schizophrenia [18].

Given these premises, we carried out a *GRN* association study in patients with BPD and schizophrenia compared with controls. In addition, we measured progranulin plasma levels and correlated them with genetic data.

## Results

Genetic variation within *GRN* was analyzed in a German population of 508 patients with schizophrenia and BPD as compared with 574 matched controls (Table 1). Both control and case populations were in HWE for all SNPs studied. Considering each SNP alone, a significantly decreased allelic frequency of the minor versus the wild-type allele was observed for rs2879096 (23.2 versus 34.2%, *P*<0.001, OR:0.63, 95%CI:0.49–0.80, Table 2), rs4792938 (30.7 versus 39.7%, *P* = 0.005, OR: 0.70, 95%CI: 0.55–0.89, Table 2) and rs5848 (30.3 versus 36.8%, *P* = 0.007, OR: 0.71, 95%CI: 0.56–0.91, Table 2). For all three polymorphisms, there seemed to be an additive effect in that the effect was stronger when carrying two polymorphic alleles (see details in Table 2). Stratifying patients according to the diagnosis, a significant association could still be seen in both schizophrenia and BPD (Table 2). Regarding haplotype analysis, none of the selected SNPs were in strong LD (D' ranging from 0.1 to 0.9, data not shown). Accordingly, none of upper CI values met the criteria for haplotype analysis according to the method used [19].

**Table 1:** Characteristics of German individuals included in the association study.

	CON	CASES		
			Schizophrenia	BPD
n	574	508	271	237
Gender (M∶F∶unknown)	230∶341∶3	229∶273∶6	146∶120∶5	83∶153∶1
Mean age, yrs±SEM (range)	27.4±0.39 (18–68)	28.30±0.47 (9–72)*	27.13±0.59 (9–72)*	30.14±0.76 (14–63)*

* **age at disease onset.**

**Table 2:** Allelic and genotype frequencies given as %(n) in German cases compared with age-, gender- and ethnicity matched controls.

SNP	n°	Genotype % (n)			Allele % (n)	
**rs2879096**		*CC*	*CT*	*TT*	*C*	*T*
Controls	574	46.7 (268)	38.2 (219)	15.1 (87)	65.8 (755)	34.2 (393)
BPD cases	237	59.5 (141)	37.1 (88)	3.4 (8)*	78.1 (370)	21.9 (104)**
SZ cases	271	57.6 (156)	36.5 (99)	5.9 (16)***	75.8 (411)	24.2 (131)
All cases	508	58.3 (296)	37.0 (188)	4.7 (24)****	76.8 (780)	23.2 (236)*****
**rs3785817**		*AA*	*GA*	*GG*	*A*	*G*
Controls	574	51.4 (295)	39.7 (228)	8.9 (51)	71.3 (818)	28.7 (330)
BPD cases	237	59.1 (140)	35.0 (83)	5.9 (14)	76.6 (363)	23.4 (111)
SZ cases	271	55.7 (151)	38.0 (103)	6.3 (17)	74.7 (405)	25.3 (137)
All cases	508	57.1 (290)	36.8 (187)	6.1 (31)	75.5 (767)	24.5 (249)
**rs4792938**		*GG*	*GC*	*CC*	*G*	*C*
Controls	574	38.5 (221)	43.6 (250)	17.9 (103)	60.3 (692)	39.7 (456)
BPD cases	237	48.5 (115)	43.9 (104)	7.6 (18)°	70.5 (334)	29.5 (140)°°
SZ cases	271	46.5 (126)	43.9 (119)	9.6 (26)°°°	68.5 (371)	31.5 (171)
All cases	508	47.2 (240)	44.1 (224)	8.7 (44)°°°°	69.3 (704)	30.7 (312)°°°°°
**rs9897526**		*GG*	*GA*	*AA*	*G*	*A*
Controls	574	76.3 (438)	22.3 (128)	1.4 (8)	87.5 (1004)	12.5 (144)
BPD cases	237	82.7 (196)	16.0 (38)	1.3 (3)	91.1 (432)	8.9 (42)
SZ cases	271	76.7 (208)	21.8 (59)	1.5 (4)	87.6 (475)	12.4 (67)
All cases	508	79.5 (404)	19.1 (97)	1.4 (7)	89.1 (905)	10.9 (111)
**rs5848**		*CC*	*CT*	*TT*	*C*	*T*
Controls	574	39.9 (229)	46.5 (267)	13.6 (78)	63.2 (725)	36.8 (217)
BPD cases	237	48.5 (115)	41.3 (98)	10.2 (24)	69.2 (328)	30.8 (146)
SZ cases	271	48.7 (132)	43.9 (119)	7.4 (20)•	70.7 (383)	29.3 (159)••
All cases	508	48.2 (245)	42.9 (218)	8.9 (45)•••	69.7 (708)	30.3 (308)••••

* *P*<0.001, OR: 0.20, 95%CI: 0.09–0.14.

** *P* = 0.001, OR: 0.60, 95%CI: 0.44–0.81.

*** *P* = 0.0002, OR: 0.35, 95%CI: 0.20–0.61.

**** *P*<0.001, OR: 0.28, 95%CI: 0.17–0.44.

***** *P*<0.001, OR: 0.63, 95%CI: 0.49–0.80.

° *P* = 0.0003, OR: 0.37, 95%CI: 0.22–0.63.

°° *P* = 0.01, OR: 0.66, 95%CI: 0.49–0.90.

°°° *P* = 0.002, OR: 0.48, 95%CI: 0.31–0.77.

°°°° *P*<0.001, OR: 0.43, 95%CI: 0.30–0.63.

°°°°° *P* = 0.005, OR: 0.70, 95%CI: 0.55–0.89.

• *P* = 0.01, OR: 0.51, 95%CI: 0.30–0.85.

•• *P*<0.0001, OR: 0.53, 95%CI: 0.39–0.72.

••• *P* = 0.019, OR: 0.62, 95%CI: 0.42–0.91.

•••• *P* = 0.007, OR: 0.71, 95%CI: 0.56–0.91.

**Figure 1:**
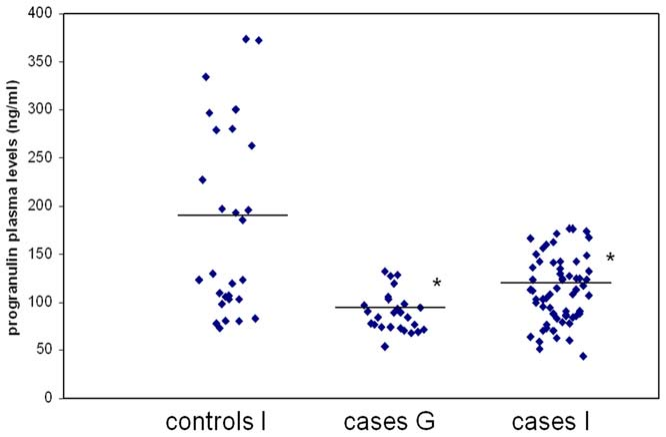
Scattergram of progranulin plasma levels in BPD patients and controls. Black lines represent mean values. G = German. I = Italian. *P<0.001, patients versus controls.

Progranulin plasma levels were measured in 26 German BPD patients. As one of them had levels below the reference range [9], *GRN* was sequenced, but no causal mutations were identified. Although exceeding the lower cut-off level, mean progranulin levels in patients were lower than previously published data obtained in Italian controls [20]. We thus evaluated progranulin levels in an independent cohort of Italian volunteers (n = 29) and compared them with German cases (Table 3), again showing a significant difference in means levels ±SEM (180,81±18.39 ng/ml in controls versus 89.69±3.97 ng/ml in patients, *P*<0.001, Figure 1). Similar levels were found also in an Italian population of 61 patients with BPD (116.14±5.80 ng/ml versus controls, *P*<0.001, Figure 1). Progranulin levels were not correlated with age either in patients (ρ = 0.07, *P* = 0.568 in the Italian population, ρ = 0.17, *P* = 0.662 in German patients, data not shown) or controls (ρ = −0.11, *P* = 0.578, data not shown). Stratification according to each of the SNPs studied, no significant differences in progranulin plasma levels were shown (data not shown).

**Table 3:** Characteristics of Italian (I) and German (G) subjects included in plasma level evaluation.

	CONI	BPDI	BPDG
n	29	61	26
Gender (M∶F)	10∶19	25∶36	9∶17
Mean age at sampling, yrs±SEM (range)	67.89±1.83(50–83)	52.35±1.63(20–64)	42.23±2.78(23–72)

## Discussion

To our best knowledge, this is the first evidence that *GRN* variability decreases the risk to develop BPD and schizophrenia. In addition, progranulin plasma levels are significantly decreased in patients as compared with controls. Despite both the SNPs and progranulin levels were associated with the target phenotypes, no association between such SNPs and progranulin levels were observed. This could be due to a number of reasons, including the possible regulation of translation by microRNA, the interaction of additional variants not included in this study, or the effect of medications taken at time of plasma sampling. Unfortunately, at time of DNA sampling, no matched plasma samples were taken from German controls. Therefore, these preliminary results need a further confirmation in a larger and ethnically matched population.

Progranulin and the various granulin peptides derived by elastase cleavage are implicated in a range of biological functions, including development, wound repair, inflammation and tumorigenesis [21]. Whereas progranulin has anti-inflammatory properties, granulins display pro-inflammatory actions [22]. Our observation that progranulin levels are low in plasma from patients with schizophrenia and BPD could imply that the balance between progranulin and granulins is altered in favour of granulins, that increase the degree of inflammation. A number of findings suggest a role for inflammatory factors in schizophrenia and BPD pathogenesis. Suvisaari et al., [23] analyzed inflammatory markers in psychotic disorders and their association with metabolic comorbidity, antipsychotic medication, smoking, alcohol use, physical condition, and mood, showing that mononuclear phagocyte system was mostly related to metabolic comorbidity and antipsychotic medication use, whereas T-cell activation had a more direct relationship with both psychotic disorders and depressive symptoms. In addition, Interleukin (IL)-6 serum levels were significantly increased in patients with schizophrenia as compared with controls, whereas IL-10 concentration was increased in both patients with schizophrenia and BPD [24].

To date, a number of Genome Wide Association Study (GWAS) have been performed in patients with either schizophrenia or BPD [25]. Among these, an association with 17q21, the locus containing *GRN*, has been shown in Latino populations with schizophrenia [18] and in patients with BPD [26]. Notably, this locus has been implicated in several other neurological and psychiatric pathologies of the central nervous system, including FTLD (see [27] for review), PSP [28], and autism [29], [30], supporting the hypothesis of common pathogenic mechanisms among these diseases. Regarding the occurrence of FTLD and schizophrenia, in support of the hypothesis that these diseases share a common aetiology, Schoder et al. [8] demonstrated that the morbid risk for schizophrenia is significantly higher in relatives of FTD probands than in relatives of Alzheimer's disease probands. In three mixed families, a causal *GRN* mutation was found, even in family members diagnosed with schizophrenia [8]. In addition, TDP43 pathology was found in patients whose first clinical presentation was consistent with schizophrenia or BPD [5]. Regarding our population, we did not have the opportunity to follow up patients and their families to ascertain the development of dementia.

In our study, the association observed in the whole population was maintained after stratifying in patients with BPD and schizophrenia, suggesting common pathogenic pathways. In line with this hypothesis, two large GWAS in BPD and schizophrenia patients, respectively, lead to the identification of the same susceptibility genes [31], [32]. Nevertheless, a replication analysis should be carried out to confirm data described here.

Regarding progranulin levels, we acknowledge that the majority of patients were treated at time of sampling, thus we can't exclude an influence of therapies on progranulin levels.

In conclusion, we demonstrated that *GRN* variability contributes to schizophrenia and BPD development and that progranulin plasma levels are lower in patients with BPD than in controls, although this findings need a replication in a larger cohort.

## Materials and Methods

### Subjects

Five hundreds and eight patients with psychiatric disorders, including 229 males, 273 females, and 6 gender unknown, mean age±SEM at sampling: 48.99±0.65 years (range 22–85), mean age±SEM at disease onset: 28.30±047 years (range: 9–72) were recruited at the Department of Psychiatry, Psychosomatics and Psychotherapy, University of Würzburg, Germany. Two hundreds seventy one patients, including 146 males, 120 females, and 5 gender unknown, mean age±SEM at sampling: 47.00±0.84 years (range: 22–85), mean age±SEM at disease onset: 27.13±0.59years (range: 9–72) were diagnosed with schizophrenia [33], whereas 237 patients including 83 males, 153 females and 1 gender unknown, mean age±SEM at sampling: 51.96±1.00 years (range: 25–85), mean age±SEM at disease onset: 30.14±0.76 years (range: 14–63) were diagnosed with BPD (type 1: n = 103, type 2: n = 78, not defined: n = 56) [34].

The control group consisted of 574 German volunteers matched for ethnic background and age (230 males, 341 females, 3 gender unknown, mean age ± SEM: 27.4±0.39 years, range 18–68) and was recruited at the Department of Psychiatry, Psychosomatics and Psychotherapy, University of Würzburg, Germany. The age of controls did not significantly differ from that of patients' age at disease onset (*P*>0.05). The control sample was composed of blood donors, staff members, and volunteers all originating from the Lower Franconia region. The sample was not systematically screened for psychiatric disorders; however, all subjects were free of medication, and the study was explained to them, so that the likelihood of severe psychiatric disorders in the control sample was low. Only subjects who gave written informed consent were enrolled in the study, which complied with the Declaration of Helsinki and was approved by the Ethics Committees of the University of Würzburg.

Plasma samples were collected in 26 German BPD patients, including 9 males and 17 females, mean age±SEM at sampling: 42.23±2.78 years (range 23–72). Additional plasma samples were collected from 61 Italian patients with BPD, including 25 males and 36 females, mean age±SEM at sampling: 52.35±1.63 years (range: 20–64) and 29 controls (10 males and 19 females), mean age±SEM at sampling: 67.89±1.83 years (range: 50–83) at the University of Milan, Policlinico Hospital, Milan, Italy. Psychiatric diagnoses were performed through the administration of a semi-structured clinical interview for a DSM-IV-TR Axis 1 disorders (SCID-I/P) [35] by trained psychiatrists.

This study has been approved by the Institutional Review board of the Fondazione Cà Granda, IRCCS Ospedale Maggiore Policlinico. Details of patients and controls are shown in Table 1.

### DNA isolation and GRN SNPs analysis

High-molecular weight DNA was isolated from EDTA blood by using a standard de-salting method. DNA samples were aliquoted and stored at −20°C until use. For the association analysis, four optimal tagging SNPs covering the *GRN* sequence were analyzed (details in [14]). In addition, rs5848, located in the 3′UTR and previously shown to influence mRNA transcription through miR-659 regulation [12], was included in the analysis as well.

Tagging SNPs were analyzed by using TaqMan methodology. Each Taqman 5′-nuclease assay employed 25 ng of genomic DNA as template. Assay-on-demand products, ABI assay IDs: C_15835934_10, C_27482034_10, C_32346749_10, C_2548248_10, C_7452046_10 were used for rs2879096, rs3785817, rs4792938, rs9897526 and rs5848 genotyping respectively. Details of the protocol used are given elsewhere [14].

### GRN mutation scanning

The entire open reading frame including the noncoding exon 0 and exon-intron boundaries of exons 1–12 of the *GRN* gene was sequenced using specific primers, as previously described [36] in one patient with progranulin plasma levels under the normality threshold [9].

### Statistical analysis

Allelic and genotypic frequencies were obtained by direct counting. Chi square test was used to test for Hardy Weinberg Equilibrium (HWE, http://www.husdyr.kvl.dk/htm/kc/popgen/genetik/applets/kitest.htm). Chi^2^ was used for differences in SNP distribution between cases and controls. Bonferroni's correction was applied. The Odds Ratio (OR) was calculated along with its 95% Confidence Interval (CI). Haploview 3.2 software was used to test for LD and for differences in haplotype distribution between cases and controls. Statistical significance was estimated empirically using the bootstrap function in Haploview. Bootstrap *P*-values are calculated using 10000 bootstrap samples. Calculation of D' is based on block definition by Gabriel et al. [19]. Progranulin levels were compared by using the Kurskall-Wallis one way analysis of variance with Dunn's method for multiple comparisons.
